# The Effects of Transcranial Direct Current Stimulation on the Cognitive Functions in Older Adults with Mild Cognitive Impairment: A Pilot Study

**DOI:** 10.1155/2018/5971385

**Published:** 2018-03-15

**Authors:** Pablo Cruz Gonzalez, Kenneth N. K. Fong, Ted Brown

**Affiliations:** ^1^Department of Rehabilitation Sciences, The Hong Kong Polytechnic University, Hong Kong, Hong Kong; ^2^Department of Occupational Therapy, Monash University, Melbourne, VIC, Australia

## Abstract

**Objective:**

The aim of this pilot study was to investigate whether the use of anodal transcranial direct current stimulation (tDCS) on the left dorsolateral prefrontal cortex could boost the effects of a cognitive stimulation (CS) programme using a tablet on five older adults with mild cognitive impairment (MCI).

**Method:**

A single-subject study of A-B-C-A design was used. After the baseline with the administration of CS (phase A), a sham treatment with CS was applied (B). Following the withdrawal of sham treatment, tDCS was introduced in combination with CS (C). Finally, phase A was replicated a second time.

**Results:**

tDCS had a significant effect on processing speed, selective attention, and planning ability tasks in terms of performance and completion time.

**Conclusion:**

tDCS appears to have a positive impact on some cognitive components in CS in persons with MCI. Further study on its long-term effects and generalization of power to daily activities is warranted.

## 1. Introduction

Mild cognitive impairment (MCI) is a syndrome of cognitive decline below the typically expected age norm in an individual. It is commonly referred to as an intermediate phase between the expected cognitive decline of normal aging and the pathological cognitive decline linked to dementia and usually does not interfere with daily activities [1]. There is a difference between MCI and a formal diagnosis of dementia: the latter represents a more severe cognitive decline and has a substantial negative impact on daily functioning [2]. In some cases, MCI will revert to normal cognition or remain stable. Only an insignificant proportion of people presenting with known MCI, 12–15% per year, will gradually worsen and develop dementia, compared to 1-2% of the general population; approximately, 40–65% of patients with MCI will eventually progress to Alzheimer's disease (AD) [1].

Regarding possible interventions to tackle MCI, there is a lack of evidence for pharmacological interventions that can prevent cognitive decline or conversion to dementia. To date, drugs have proved to have no positive impact in MCI trials [3].

As a form of nonpharmacological intervention, cognitive rehabilitation is defined as “the therapeutic process of increasing or improving an individual's capacity to process and use incoming information so as to allow increased functioning in everyday life. This includes methods to train and restore cognitive function and compensatory techniques” [4].

One type of cognitive rehabilitation is cognitive stimulation (CS) which has been used as a potential intervention to slow down the deterioration of cognitive functions in people presenting with known MCI. According to the largest randomized controlled trial of cognitive intervention carried out with older adults to date, the experimental treatment approaches used in this study support the improvement of targeted cognitive areas in different groups in comparison to the control group, which did not receive any kind of intervention [5]. Contrary to conventional cognitive tasks that are performed with paper-and-pencil with a lack of simultaneous feedback, computerized cognitive stimulation is designed to be more enjoyable and engaging based on human-computer interaction [6, 7]. These cognitive stimulation strategies have also been shown to improve performance after repetition of computerized CT tasks in older adults presenting with known MCI [8].

A systematic review found evidence of memory and executive function enhancement while analyzing the effects of nonpharmacological interventions on cognitive functions in older people presenting with known MCI [9]. However, the appropriate protocol and optimal frequency for inducing benefits in the cognitive functioning of this population remain unknown.

Transcranial direct current stimulation (tDCS) is another type of nonpharmacological intervention that uses direct electrical currents to stimulate specific parts of the brain. It involves delivering a noninvasive weak direct current (1-2 mA) through at least two electrodes, at least one of which is placed on the scalp for a period of a few seconds to 20–30 minutes, which modulates neuronal activity. There are two types of stimulation: anodal stimulation acts to excite neuronal activity and cathodal stimulation has hyperpolarizing effects, inhibiting neuronal activity [10, 11]. As soon as tDCS is administered, the current travels in an anode-cathode circuit which is likely to cause neurons to fire in stimulated areas [10].

Priming is the change in repetitive behavior due to implicit learning based on previous stimuli [12], and it has recently been used for inducing neuroplasticity and enhancing the effects of conventional rehabilitation as combined approaches [13]. The excitability modulation induced by tDCS is considered a potential intervention to modulate the learning processes [14]. tDCS boosts subthreshold neuronal action potentials beyond their unaugmented state, thus, may achieve stronger firing patterns than would occur in the absence of tDCS. Although, repeated practice with cognitive stimuli in CS may elicit unintentional learning, mechanisms that circumvent cognitive impairments, targeting a neural circuit with tDCS whereas it is simultaneously engaged by a cognitive stimulation task, may produce better therapeutic effects than stimulating the same cortical area in the absence of cognitive stimuli [15, 16]. tDCS may augment the strength of transmission across synaptic circuits in pathways that are stimulated by cognitive practice, and thus it may also strengthen the circuits that are formed through unintentional, practice-related learning and maximize the possibility of enduring behavior change through such implicit learning. Given that CS and tDCS can enhance plastic changes, the combination of both techniques could cause a better synergistic positive effect on behavior [15, 17]. Indeed, it has been shown that anodal stimulation of the left dorsolateral prefrontal cortex (DLPFC) increases the performance of a sequential-letter working memory task in healthy young adults [18]. Recent research also indicates that healthy older adults can benefit from tDCS, enhancing retention skills of object-location learning a week after completion of the object-location task compared to participants who took part in a tDCS sham condition [19]. There is growing evidence that tDCS coupled with CS improves cognitive performance. After ten sessions of a working memory CS in combination with tDCS, healthy adults experience an enhanced effect and perform CS tasks more accurately than those who received sham tDCS [20].

The impact of tDCS has also been explored for AD, frontotemporal dementia, and mild vascular dementia. Positive effects were found in visual recognition memory tasks in persons with AD when applying anodal tDCS to the left temporal cortex [21]. Results after five consecutive sessions over five days in which anodal tDCS was applied over both hemispheres of the temporal cortex and an extracephalic cathodal tDCS (for a 30-minute period using 2 mA) showed significant improvement in the performance of a visual recognition memory test [22]. In a more recent study that involved participants presenting with mild vascular dementia, four consecutive day sessions of anodal tDCS (for a 20-minute period using 2 mA) on the left DLPFC generated positive additional effects on visual short-term memory, verbal working memory, and executive control [23].

The beneficial effects of tDCS on cognition in people presenting with known MCI have been demonstrated [24]; however, the literature on using tDCS on people presenting with known MCI is still very limited. The frequency and targeted areas are not the only significant issues that remain unknown. To optimize the positive and therapeutic benefits of noninvasive brain stimulation (NIBS), it is also worth investigating the uncertainty of combining tDCS with conventional behavioral treatments such as a CS that might also yield more information and understanding about the impact of tDCS effects for people at risk of MCI.

Based on the above background information, we considered the use of anodal tDCS on the left DLPFC (30 minutes 2 mA) with an extracephalic return electrode to be a promising and safe intervention approach to optimize the impact of CS on tablet PCs for older adults at risk of MCI. The current study aimed to compare the impact of anodal and sham tDCS applied to the left DLPFC on the cognitive performance of people at risk of MCI engaging in CS interventions on tablet PCs. We hypothesized that there would be a significant improvement in cognitive task performance after the use of tDCS, which would subsequently generalize to other cognitive domains—short-term memory, planning ability, working memory, attention, and processing speed skills. We also aim to determine the optimal frequency of tDCS application with the same dosage to improve the cognitive skills of older adults with MCI.

## 2. Materials and Methods

### 2.1. Participants

Five older adults with MCI were recruited by convenience sampling from community center groups in Hong Kong. The inclusion criteria followed the modified Petersen's criteria [25] (given by the MCI Working Group of the European Consortium on Alzheimer's Disease, Brescia Meeting, Italy, June 2005). Participants had to (a) be aged between 60 and 85; (b) obtain a score between 19 and 26 on the Montreal Cognitive Assessment Test (MoCA) [26]; (c) achieve a score of 0.5 or below on the clinical dementia rating (CDR) [27]; (d) self-report cognitive decline; (e) be independent in daily living activities; and (f) have completed three or more years of primary education.

Regarding exclusion criteria, the following were excluded: (a) individuals presenting with a diagnosis of dementia or any other neurological disease and mental disorders; (b) individuals with depression, determined by a score of 5 or above on the Geriatric Depression Scale (GDS) [28]; and (c) individuals who had metallic fixtures around the cephalon.

The study was conducted in accordance with the Declaration of Helsinki and was approved by the human subject ethics committee of The Hong Kong Polytechnic University (ref. number: HSEARS20160415002). All participants gave informed written consent before the intervention began.

### 2.2. Design

**Figure 1:**
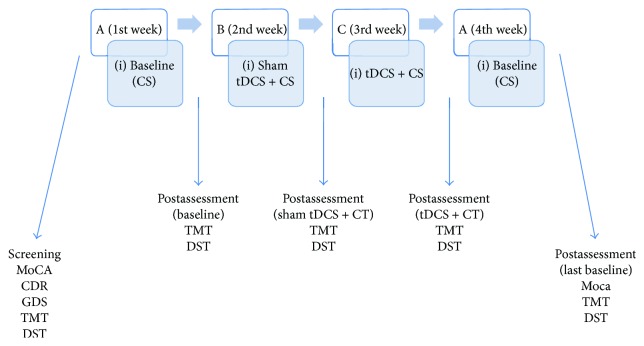
Intervention sequence. A-B-C-A design; MoCA: Montreal Cognitive Assessment; CDR: Clinical Dementia Rating; GDS: Geriatric Depression Scale; TMT: Trail Making Test; DST: digit span test. Phases. CS: cognitive stimulation; tDCS: transcranial direct current stimulation.

This study utilized a prospective, single-subject design (SSD) with multiple nonconcurrent treatments—anodal tDCS + CS, sham tDCS + CS, and CS only. A four-phase A-B-C-A SSD was employed. After the baseline with the administration of CS (phase A), a sham tDCS with CS was applied (B). Following the withdrawal of this sham treatment, a tDCS treatment was introduced in combination with CS (C). Finally, phase A was replicated to provide the control needed to document the differences between the sham and tDCS phases (Figure 1).

In this design, it is assumed that both treatments—B and C—have differential and independent effects. Differences in the target responses are expected across the four phases of the study. The sham phase (B) was the first treatment intervention to be used to avoid possible carryover effects due to the tDCS stimulation treatment (i.e., phase C), thereby eliminating this potential treatment effect, which can be analyzed during the last baseline (A).

### 2.3. Cognitive Stimulation

“Neuron Up” was the CS administered to participants. It is a web platform (https://www.neuronup.com/) designed to serve as a fundamental support for professionals involved in cognitive rehabilitation and cognitive stimulation [29]. The display format was full screen in a 9.7-inch screen iPad situated on a desk approximately 35 centimeters in front of the participant.

Participants' individualized level was identified through two training sessions that were conducted for all the participants prior to the implementation. Five cognitive activities associated with different cognitive domains were selected:

- Sorting bugs: This task is associated with planning ability and divided attention. Participants are asked to move a bar located in the middle of the screen either to block the movement of bugs which are moving in different directions or to let them pass from one side to the other. The final goal is to keep the green bugs on the green side and the red bugs on the red side. Participants are allowed seven minutes to complete the task, and the completion time is measured. This task also trains sustained and selective attention.
- The last light on: This task is associated with processing speed and selective attention. Participants are asked to pay attention to the windows in a building that light up. They have to touch the window which is the last to light up. This task is repeated five times per session, and the number of correct answers and completion time are measured.
- Illuminated windows: This activity is associated with short-term memory. Participants are asked to remember which windows are illuminated in a building in an open memorization period. Then, all the lights are turned off and participants must identify the windows that had been lit. This activity is repeated five times. The number of correct answers, number of errors, memorization time, and completion time are measured.
- Addition and subtraction questions. Both tasks are associated with calculation and working memory. Participants are given three addition operations involving four numbers of six digits each and six subtraction operations with two numbers of six digits each to solve. The number of errors and the completion time are measured.

These five cognitive activities were presented as a cognitive stimulation practice with one-to-one supervision from an occupational therapist in which all participants were exposed to repetitive testing via the computer system across sessions.

### 2.4. tDCS

The Soterix Medical 1 × 1 low-intensity tDCS stimulator was the device used to provide the stimulation. The two rubber electrodes employed for tDCS in this study were introduced in saline-soaked synthetic sponges (7 × 5 cm, 35 cm^2^).

Anodal tDCS was delivered to the left DLPFC, and the cathode electrode was placed over the contralateral deltoid muscle as extracephalic cathode. The scalp electrode was positioned over F3 according to the 10–20 EEG international system. The left DLPFC was targeted as the stimulation site because of its role in high-order cognitive processes [30] and due to the existence of functional disconnection of the DLPFC in persons with MCI [31]. A constant current of 2 mA was applied for 30 minutes. For sham tDCS, the 2 mA intensity was only given for 30 seconds at the beginning and the end of the stimulation.

### 2.5. Experimental Protocol and Procedures

Each interval (A, B, C, and A) was staggered by a week at a time. During the baseline phases, three sessions of CS were implemented for all the participants. Both interventions, sham tDCS and anodal tDCS, were combined with the same CS that was performed for the baseline phases. However, the treatment phases varied from one to five sessions. The sessions per phase were distributed over five days. Participants were randomly assigned to combinations of intervention each of which had a different time span to compare the treatment frequency effect (Table 1).

The experimental sessions were 30 minutes in length. In this way, tDCS was administered for 30 minutes and the CS was begun five minutes after the tDCS began, thus running for 25 minutes. For the sham phase, the administration of the sham tDCS lasted 30 minutes too, with the difference that a ramping current of 2 mA was applied during the first and last 30 seconds. The participants remained blinded for both stimulation conditions.

### 2.6. Cognitive Measures

CS data were recorded for each task of each cognitive activity during the sessions. Data such as completion time and performance in terms of correct answers or number of errors were collected.

The standardized cognitive assessments used in this study for screening were the CDR (Hong Kong Version), and the scale was found to have good reliability with internal consistency ranging from 0.7 to 0.9 [32], the GDS-15 item (Hong Kong version) which has a satisfactory reliability with Cronbach alpha = 0.82 [33], and the MoCA (Hong Kong Version) with a sensitivity of 90% to detect MCI [26, 34].

The standardized cognitive measures to assess the study phases included the MoCA (Hong Kong version) [34], the digit span test (DST) [35], and the Trail Making Test ((TMT) Chinese version) which normative data has provided evidence that the part B (Chinese version) may be equivalent to the standard part B [36].

The participants were assessed in five phases: screening (pre-A), after baseline (post-A), after first intervention (post-B), after second intervention (post-C), and after final baseline (post-A).

To summarize, DST and TMT were conducted before the initial baseline and after each interval. However, the MoCA was only administered before the first baseline and after the last for a general comparison of the whole sequence and to avoid learning effect due to repeated testing (Figure 1).

### 2.7. Data Analysis

To study the effects of tDCS on the “Neuron Up” CS program across the design phases, visual analysis and two standard deviation procedures were used as analytical methods.

Visual analysis was based on observing the visual patterns presented in the graphs where the target parameter changed once the treatment was introduced or withdrawn. Difference in means among phases was also compared.

In the two standard deviation procedure, the levels of the baseline are compared to those of the intervention data points. The procedure assumes that if we are to extend the baseline, then ultimately 95 percent of our observations would be less than two standard deviations away from the baseline mean. The two standard deviations were calculated manually following the guidelines set out by Rubin and Babbie [37]. Data analyses of the cognitive assessments administered before the commencement of the baseline and after every single interval were compared.

## 3. Results

Although all five participants did well in the tDCS intervention, redness in the area was observed after removing the electrodes in one participant, and he also complained of having a mild headache a few hours after receiving the therapy. The remaining participants reported a tingling sensation in the DLPFC region during the stimulation phase which faded away after a few minutes of the onset of the stimulation. They completed all sessions as scheduled, with the exception of one participant who was not available to complete the last session of the last baseline.

### 3.1. Cognitive Stimulation Outcomes

The results are presented in graphs in the sequence in which the CS tasks were performed and following the order from fewer to more treatment sessions received. The *x*-axis corresponds to the observation points (the number of tasks) per day. The *y*-axis represents either the performance or the time taken to complete the task. The blue line is the measurement of the targeted problem across observation points. There were four intervals for each condition: (A) baseline, (B) sham tDCS intervention, (C) tDCS intervention, and (A) baseline. Every single black line which crosses every interval is the mean of the performance, and the two standard deviations are marked by a black dotted line starting at the corresponding interval.

**Figure 2:**
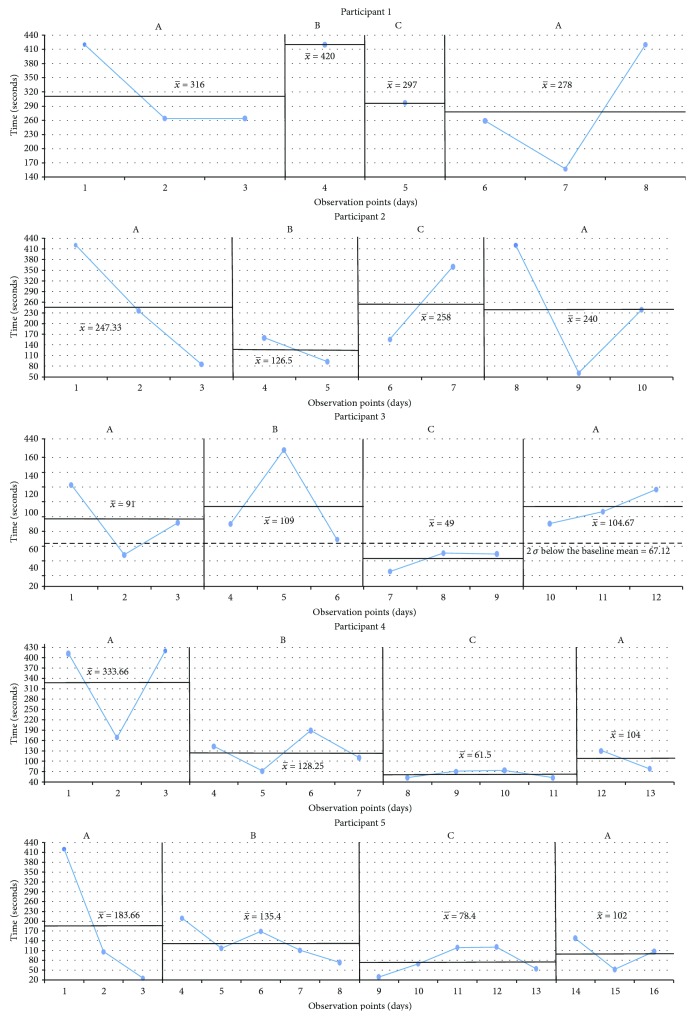
Sorting bugs. A: baseline; B: sham tDCS; C: tDCS; *x*-axis: observation points in days; *y*-axis: completion time in seconds. Scores are shown, along with black lines marking the average of each phase and with a black dotted line starting at the corresponding baseline marking 2-standard deviation (2 *σ*) when there is statistically significant difference (participant 3).

**Figure 3:**
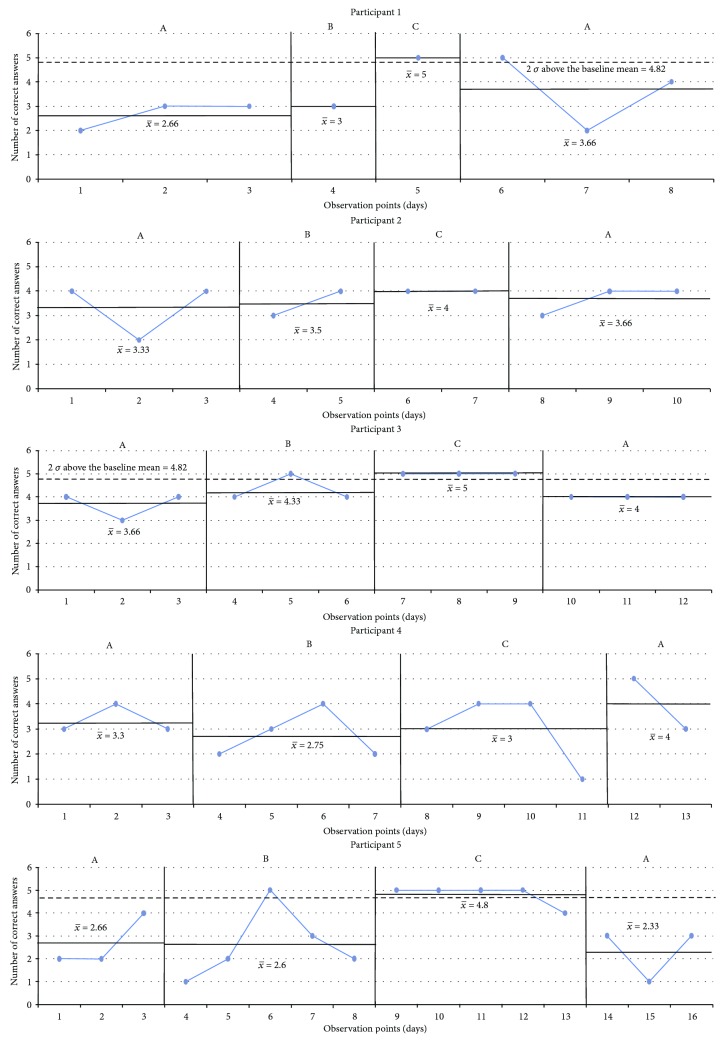
The last light on. A: baseline; B: sham tDCS; C: tDCS; *x*-axis: observation points in days; *y*-axis: number of correct answers. Scores are shown, along with black lines marking the average of each phase and with a black dotted line starting at the corresponding baseline marking 2-standard deviation (2 *σ*) when there is statistically significant difference (participants 1, 3, and 5).

**Figure 4:**
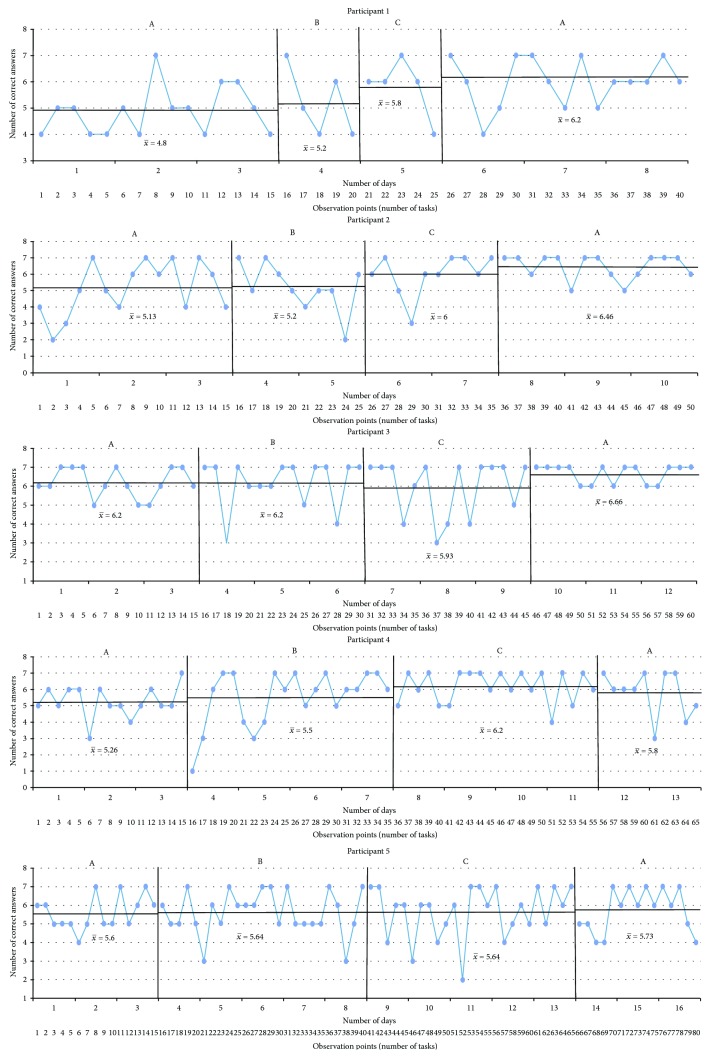
Illuminated windows. A: baseline; B: sham tDCS; C: tDCS; *x*-axis: observation points in number of tasks performed within days; *y*-axis: number of correct answers. Scores are shown, along with black lines marking the average of each phase.

**Figure 5:**
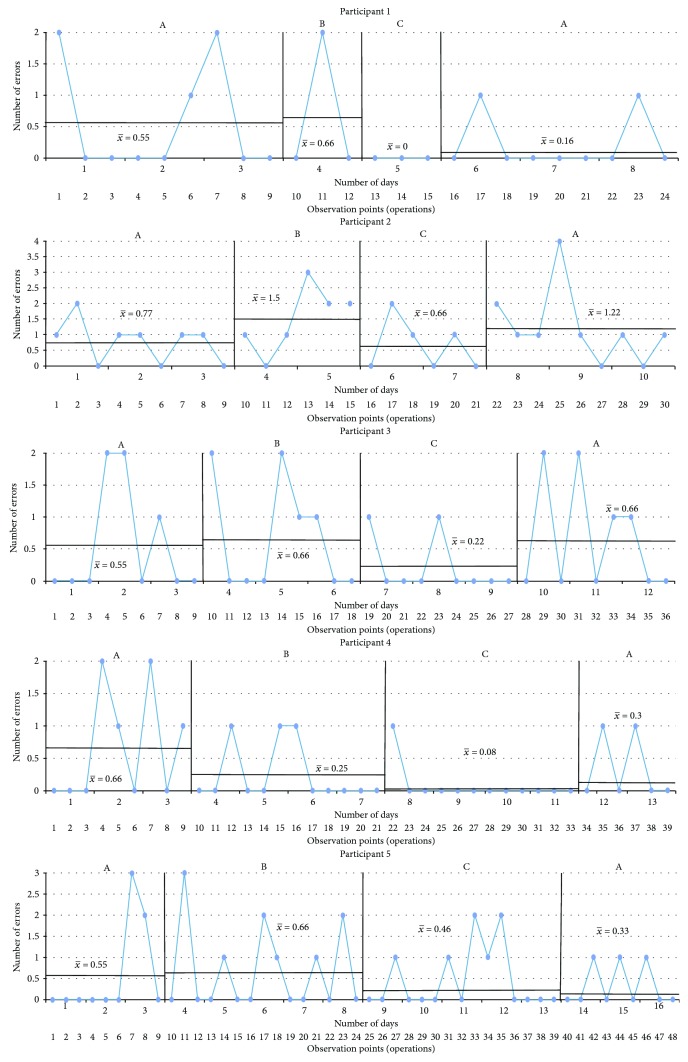
Additional question. A: baseline; B: sham tDCS; C: tDCS; *x*-axis: observation points in number of tasks performed within days; *y*-axis: number of errors. Scores are shown, along with black lines marking the average of each phase.

**Figure 6:**
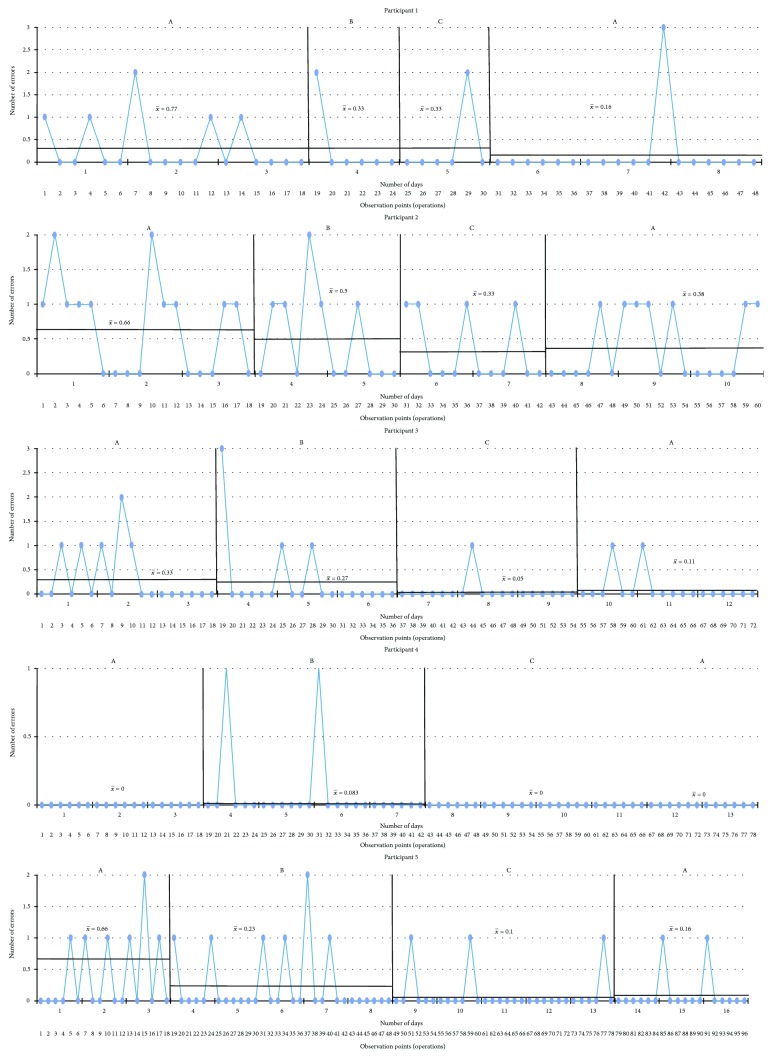
Subtraction question. A: baseline; B: sham tDCS; C: tDCS; *x*-axis: observation points in number of tasks performed within days; *y*-axis: number of errors. Scores are shown, along with black lines marking the average of each phase.

- Sorting bugs (Figure 2): All participants demonstrated fluctuating times of completion during the first baseline phase. There are positive effects for those subjects who received three or more tDCS sessions (participants 3, 4, and 5) with a general slight increase in time required to complete the task after withdrawal of the tDCS intervention, and a difference by more than two standard deviations was observed at the last baseline phase of participant 3.
- The last light on (Figure 3): Figure 3 shows that there were differences by more than two standard deviations in participants 1, 3, and 5. With respect to the baselines and sham phases, all participants exhibited decreasing accuracy in the cognitive task in comparison with the experimental interval, except for participant 4, but no significant difference was found.
- Illuminated windows (Figure 4): Despite all participants exhibiting similar outcomes in all phases, there is a slight general improvement in task performance across conditions, but no significant difference was found.
- Additional question (Figure 5): Participants 1, 2, 3, and 4 demonstrated a clear intervention effect of tDCS administration, but no significant difference was found. Participants made fewer errors in operations when the tDCS was applied. However, participant 5 performed differently, reducing the number of errors after the sham tDCS intervention and especially achieved the best performance during the last baseline phase.
- Subtraction question (Figure 6): The outcomes of these operations were similar to the additional questions, but the change in level was not very pronounced. Participants 2, 3, and 5 were more accurate, solving the operations during the tDCS treatment, and the tendency during the baseline and sham phases was associated with a larger number of errors, but no significant difference was found. For participants 1 and 4, the results were almost identical across conditions.

### 3.2. Behavioral Assessment Outcomes

#### 3.2.1. MoCA Test

All participants showed an improvement in MoCA scores. Participant 3 showed the largest improvement (Table 1).

#### 3.2.2. Trail Making Test

Participants 1 and 4 demonstrated the greatest impact of the tDCS as revealed by the shortest completion time (parts A and B) right after the last session of the tDCS intervention. The negative ratio shown in Table 2 indicates a shorter time taken to complete the task after tDCS relative to sham tDCS. Participant 3 also improved during phase B and participant 5 during phase A (Table 2).

#### 3.2.3. Digit Span Test

All participants improved in their digit span test scores when comparing the baseline to the last assessment. The trend shows that improvement follows a general and steady progressive pattern without obvious significant changes (Table 3).

## 4. Discussion

This pilot study combined anodal tDCS with CS to investigate their impact on the cognitive performance of older adults with MCI. The result shows that application of anodal tDCS to the left DLPFC and cathodal tDCS to the right deltoid muscle helps to enhance cognitive performance in processing speed, selective attention, and working memory activities, as well as the completion time in planning ability and divided attention tasks. One of the objectives of this study was to compare anodal tDCS and sham tDCS. Although the data generated with CS fluctuated and were variable, the participants did not show significantly better outcomes in the sham intervention than the baseline CS alone.

This was the first study of its kind to show mild benefits in multiple domains of cognition in older adults with MCI as other studies have focused on the possible benefits of tDCS in a single cognitive domain, usually working memory [13, 18, 38].

Placement of an anodal tDCS on the left DLPFC and a cathodal tDCS on the right deltoid muscle did not increase participants' performance in the short-term memory CS task. This agrees with previous studies that applied the same montage as the current study in combination with memory training in persons suffering from AD and which also observed no significant additional effect of tDCS on memory performance beyond that of sham tDCS with the same memory training [39].

Our study adopted extracephalic cathodal tDCS, which eliminated the confounding effect of a monocephalic cathode electrode placed on the scalp. Our findings are also in line with the study conducted by Boggio and colleagues [22] in which the return electrode was extracephalic and placed over the right deltoid muscle in people presenting with AD. The use of a monocephalic cathode setup has been controversial because “current flow direction/electrical field orientation relative to neuronal orientation might determine the effects of tDCS and it might be that the effects of an extracephalic electrode differs relevantly from that of a bipolar electrode arrangement” [40]. Monocephalic cathodes are also common in studies, but that does not mean that the return electrode is physiologically inert, since its positioning does have a critical impact on the electrical field orientation [13]. Notwithstanding, we are confident that the electrical current passes through the stimulated brain area—the left DLPFC—when applying tDCS. With the same cathode montage, both our study and that of Boggio and colleagues [22] indicate a significant improvement in visual recognition after the administration of multisession tDCS.

It is disappointing that all these positive CT findings are somewhat inconsistent with the results of standardized cognitive assessments, except for the TMT, in which most of the participants showed their best score of the tDCS intervention in all phases. Interestingly, TMT could be an indicator of processing speed [41] and visual selective attention domains [42], which might also correspond to the CS task improvement associated with these domains.

Despite our aim to determine the optimal frequency of tDCS application with the same dosage by modifying the number of sham tDCS and tDCS sessions among participants, the findings appear to be inconclusive. In some occasions, just one session of tDCS was sufficient to produce positive changes in performance while other participants who had up to five sessions of tDCS showed no evidence of benefiting from exposure to the tDCS intervention. Comparison of participants' individual performance of all the CS tasks indicates that the most beneficial dose of tDCS seems to be three sessions per week. However, conclusions cannot be gleaned from the session's variability due to the small sample of this study. This should be addressed in the future as it remains unclear.

Although this study has produced encouraging results, it also has several limitations. First, an A-B-C-A SSD was used without randomization among experimental conditions. The same order was used for all the participants because if tDCS was administered in phase B right after the baseline, then it could have affected the outcomes under the sham tDCS phase due to possible carryover effects of tDCS stimulation; therefore, it could have also disguised the sham effect we originally aimed to compare with real tDCS. This could have given rise to a second limitation during the last baseline A, either due to a training effect of the CS or a carryover effect of the tDCS administration in phase C, which cannot be separated for interpretation. This is a disadvantage of using an SSD in cognitive studies. We intended to monitor daily response in behavioral terms to different treatments, but the frequency of the application of CS in some of the participants in such a short period made it problematic to decouple what participants might have achieved by continued testing from what was changed by tDCS.

For the same reason, our original intention was to observe whether the CS outcomes could match the cognitive assessment score in every condition. To check this possibility, we administered a battery of assessments five times over a four-week interval, which might provide a learning effect and reduce overinterpreting the CS task outcomes by making a linkage with the standardized cognitive evaluations, and alternative forms of cognitive assessments to measure changes over time should be used.

Despite the limitations of this pilot study, it is essential to conduct pilot studies with NIBS techniques before the implementation of larger trials. The strength of this study allow us to monitor the daily cognitive response of single or coupling therapies gathering valuable data that can shape a future robust intervention. The ultimate purpose of using NIBS is to prove if it can be used as a feasible nonpharmacological therapy in couple with conventional treatment, in this case computer CS, for older adults with MCI. The emerging application of tDCS as a therapeutic intervention gives us the obligation of conducting studies to develop treatment programmes which can support evidence base and determine the future use of these innovative techniques in the field of cognitive rehabilitation.

## 5. Conclusion

The current study investigated the effects of anodal tDCS on CS in older adults with MCI and found mild beneficial effects on processing speed, selective attention, planning ability, and working memory which were better than those achieved by CS alone or by sham tDCS. The optimal frequency of tDCS administration remains unclear.

Further research is required to improve understanding of the neuromechanism and to determine the behavioral effects of tDCS on CS in a larger multicentered, randomized controlled study to determine the possibility of transferability to everyday cognition.

## Figures and Tables

**Table 1:** Demographics, inclusion criteria scores, and number of sessions conducted in every interval by week.

Participant	Demographics			Inclusion assessment scores					Number of sessions conducted in every interval by week			
	Age	Gender	Medical history	MoCA preintervention	MoCA postintervention	MoCA gain (%)	CDR	GDS	1st week (A) CS alone	2nd week (B) sham tDCS + CS	3rd week (C) anodal tDCS + CS	4th week (A) CS alone
1	79	Female	Heart disease	24	26	6.66	0.5	3	3	1	1	3
2	68	Female	NA	24	25	3.33	0.5	1	3	2	2	3
3	67	Male	NA	24	29	16.6	0.5	1	3	3	3	3
4	69	Male	Diabetes	25	26	3.3	0.5	1	3	4	4	2
5	81	Male	Diabetes	26	27	3.3	0.5	2	3	5	5	3

MoCA: Montreal Cognitive Assessment; CDR: Clinical Dementia Rating; GDS: Geriatric Depression Scale; NA: not applicable; CS: cognitive stimulation; sham tDCS + CS: transcranial direct current stimulation + CS during the A, B, C, and A phases.

**Table 2:** Trail Making Test score.

Participant	Trail Making Test	Baseline	After first baseline (A1)	After sham tDCS (B)	After tDCS (C)	After last baseline (A2)	Immediate effect (seconds) (C versus baseline)	After-effect (seconds) (A2 versus C)	tDCS versus sham tDCS (seconds) (C versus B)
1	Part A	58.82	58.45	51.53	44.48	55.69	−14.34	10.81	−6.82
	Part B	109.74	87.22	67.7	60.52	67.45	−49.22	6.93	−7.18
2	Part A	38.46	38.4	32.92	41.34	28.03	2.88	−13.31	8.42
	Part B	55.45	83.04	75.73	56.55	64.77	1.1	8.22	−7.31
3	Part A	26.35	22.68	22.91	20.86	15.53	−5.49	−5.53	−2.05
	Part B	42	44.92	29.68	26.73	27.13	−15.87	0.4	−3.25
4	Part A	32.48	40.16	56.12	28.08	51.93	−4.4	23.85	−28.04
	Part B	51.6	56.23	51.5	42.35	56.74	−9.25	14.39	−9.15
5	Part A	37.58	50.4	43.76	38.48	46.55	−0.9	8.07	−5.28
	Part B	57.42	40.55	71.42	52.65	50.15	−4.77	−2.5	−18.77

Immediate effect (C versus baseline) is the gain in seconds after the application of tDCS compared with the baseline; after-effect (A2 versus C) is the maintenance or gain in seconds after tDCS withdrawal in phase C; tDCS versus sham tDCS (C versus B) is the comparison between tDCS and sham tDCS. A positive ratio implies decrement, a neutral ratio implies maintenance, and a negative ratio implies improvement in terms of time of completion.

**Table 3:** Digit span test score.

Participant	Digit span test	Baseline	After first baseline (A1)	After sham tDCS (B)	After tDCS (C)	After last baseline (A2)	Immediate effect (%) (C versus baseline)	After-effect (%) (A2 versus C)	tDCS versus sham tDCS (C versus B)
1	Forward score	15	16	16	15	16	0	3.33	−3.33
	Backward score	6	8	10	8	9	6.66	3.33	−6.66
	Total score	21	24	26	23	25	6.66	6.66	−9.99
2	Forward score	14	15	14	15	16	3.33	3.33	3.33
	Backward score	5	5	7	6	8	3.33	6.66	−3.33
	Total score	19	19	21	21	24	6.66	9.99	0
3	Forward score	14	16	16	16	16	6.66	0	0
	Backward score	9	9	9	9	13	0	13.32	0
	Total score	23	25	25	25	29	6.66	13.32	0
4	Forward score	16	16	16	16	16	0	0	0
	Backward score	7	8	5	8	8	3.33	0	9.99
	Total score	23	24	21	24	24	3.33	0	9.99
5	Forward score	13	16	16	16	16	9.99	0	0
	Backward score	4	4	5	7	5	9.99	−6.66	6.66
	Total score	17	20	21	23	22	19.98	−3.33	6.66

Immediate effect (C versus baseline) is the gain (%) after the application of tDCS compared with the baseline; after-effect (A2 versus C) is the maintenance or gain (%) after tDCS withdrawal in phase C; tDCS versus sham tDCS (C versus B) is the comparison in terms of gain (%) between tDCS and sham tDCS. A positive ratio implies improvement, a neutral ratio implies maintenance, and a negative ratio implies decrement in terms of accuracy.
